# Association of TyG index and central obesity with hypertension in middle-aged and elderly Chinese adults: a prospective cohort study

**DOI:** 10.1038/s41598-024-52342-7

**Published:** 2024-01-26

**Authors:** Yang Chen, Peng Hu, Yangyang He, Hao Qin, Longlong Hu, Renqiang Yang

**Affiliations:** 1 Department of Cardiology, The Second Affiliated Hospital of Nanchang University, Nanchang, Jiangxi China; 2 Department of Blood Transfusion, The Second Affiliated Hospital of Nanchang University, Nanchang, Jiangxi China

**Keywords:** Physiology, Biomarkers, Cardiology, Diseases, Endocrinology, Medical research, Risk factors

## Abstract

Triglyceride glucose index (TyG) and waist circumstance have been well documented to be highly correlated with hypertension. However, the joint effect of waist circumstance and TyG on the risk of hypertension is unknown in middle-aged and elderly Chinese adults. The purpose of this study was to investigate the association between TyG and the risk of new-onset hypertension in middle-aged and elderly Chinese individuals with different waist circumstances. The multicentred prospective cohort study was conducted in 28 provinces of China including a total of 5865 eligible participants aged ≥ 45 years old. Cox regression was performed to examine the relationship of TyG index and hypertension with adjustments for the pertinent variables. Besides, the relationship was explored in different groups on the basis of waist circumstance. There was no significant correlation between TyG index and new-onset hypertension after adjustment for pertinent variables (hazards ratio [HR]: 0.99; 95% confidence interval [CI]: 0.80–1.24). When the association was explored in different waist circumstance groups, multivariate cox regression analyses revealed that TyG was an independent factor positively associated with the risk of hypertension in central obesity prophase group (HR: 1.57; 95% CI 1.13–2.16). Among individuals with central obesity, relative to population with lower TyG (Q1: 4.96–8.18), people who had higher TyG (Q3: 8.52–8.95; Q4: 8.95–12.14) were associated with significantly lower HR for hypertension. There was no conspicuous correlation between TyG index with new-onset hypertension in normal waist circumstance (HR: 1.05; 95% CI 0.84–1.30). The research demonstrated the positive relationship of TyG with risk of hypertension among individuals with central obesity prophase, negative relationship of TyG with hypertension among population with central obesity and inconspicuous correlation of TyG with hypertension among individuals with normal waist. In conclusion, the study findings supported the combined effects of TyG index and waist circumference in predicting hypertension in middle-aged and elderly Chinese individuals.

## Introduction

Hypertension has long been a question of great interest in a wide range of fields. It is reported that there are about 1.39 billion hypertensive patients worldwide, and the number is expected to reach 1.56 billion by 2025^1,2^. Hypertension is also a risk factor for coronary atherosclerosis, stroke, aortic dissection and other cardiovascular and cerebrovascular diseases^3,4^. About 66% of strokes and 17–22% of acute myocardial infarctions in the Chinese population are attributable to hypertension^5,6^. Identifying early predictors for hypertension is crucial for reducing its global incidence, mortality, and associated complications^7–9^.

Insulin resistance (IR), referred as decreased insulin sensitivity, is a fundamental abnormality of type 2 diabetes and an important risk factor of hypertension^10^. IR could induce activation of the sympathetic nervous system, intensified renin–angiotensin–aldosterone system (RAAS) and renal actions that promote reabsorption of sodium, leading to blood pressure elevation^11^. So far, hyperinsulinemic–euglycemic clamp is regarded as the gold diagnostic standard of IR, but only used for scientific research and cannot be widely used in clinical practice. Recently, it has been proposed that triglyceride-glucose (TyG) index is a new simple surrogate variable to identify IR in healthy individuals^12–14^.

Given the practicality of the Triglyceride-glucose (TyG) index in clinical settings, its association with hypertension has been the subject of various studies^15–17^. However, findings on the relationship between the TyG index and the incidence of hypertension have been inconsistent across different populations. Central obesity, known to be linked with insulin resistance, appears to provide additional predictive value for hypertension over general obesity^18–21^. Furthermore, research is scarce regarding the uniformity of the TyG index's predictive accuracy for hypertension among groups with differing waist circumferences. Notably, older adults are generally more prone to insulin resistance and its associated complications compared to their younger counterparts^22^. In light of these considerations, the current study was designed to assess the relationship of TyG index with the incidence of hypertension and further assess the possible joint effect of waist circumference and TyG on the risk of hypertension in middle-aged and elderly Chinese individuals.

## Methods

### Study participants

**Figure 1:**
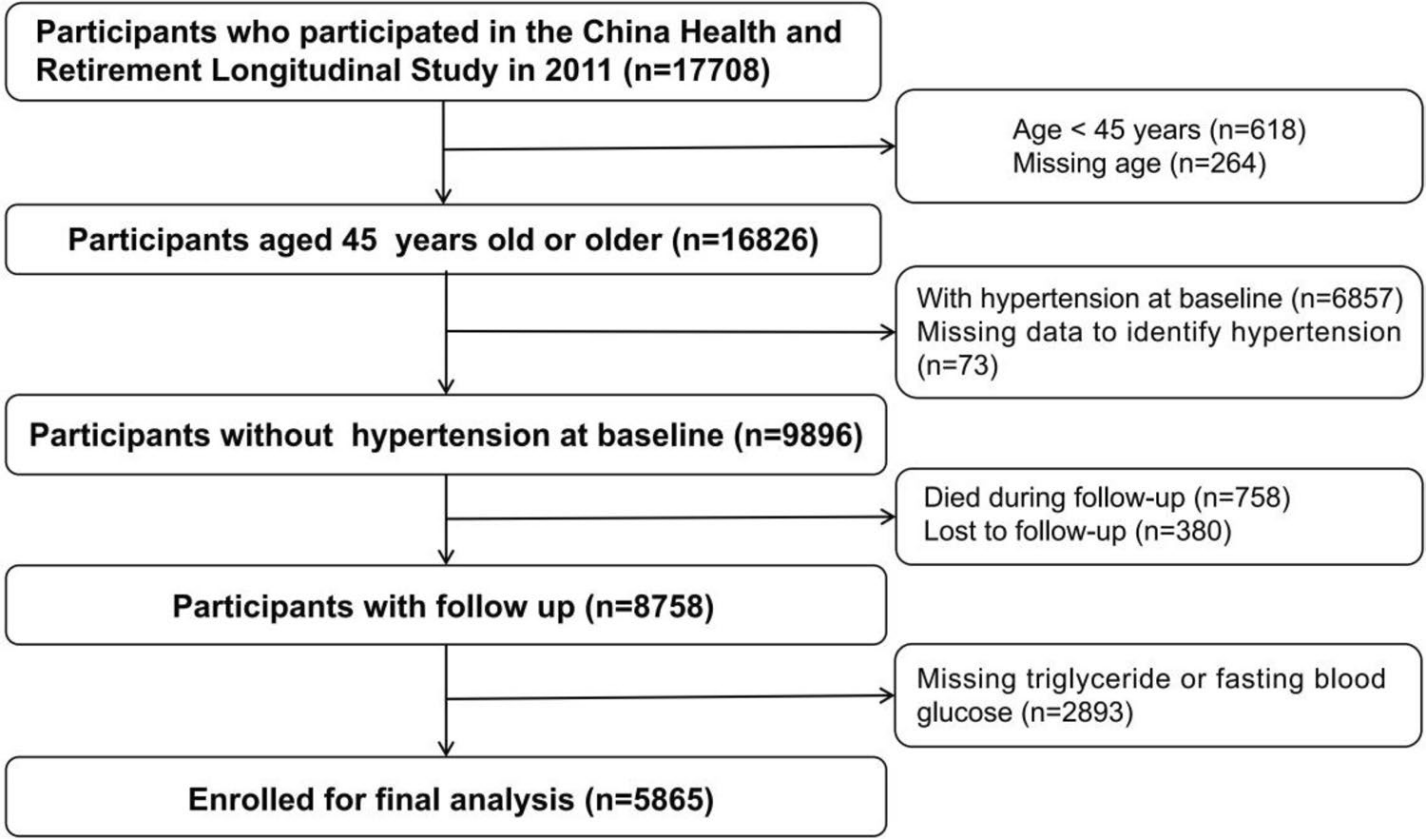
Flow diagram of study participants.

Participants were from a restricted subset of the China Health and Retirement Longitudinal Study (CHARLS). The details of data collection and the exclusion criteria have been described elsewhere^23,24^. In brief, the CHARLS applied the multistage probability sampling method, investigating 17,708 individuals through random selection of 10,257 households included from 28 provinces of China. Eligible participants were adults aged 45 years and older with a response rate of 80.5%. The data used in this research were from the CHARLS baseline survey (wave 1) in 2011–2012 and the conducted follow-up survey involving the second wave (W2) in 2013, the third wave (W3) in 2015 and the fourth wave (W4) in 2018^23^. As a result, a total of 9896 participants aged 45 years or older without hypertension were enrolled at baseline. Participants who died during follow up (n = 758), lost to follow up (n = 380) or with missing triglyceride or fasting blood glucose (n = 2893) were also excluded. Finally, 5865 subjects were included for analysis (Fig. 1). The protocol of CHARLS was authorized by the Ethics Review Committee of Peking University (Beijing, China) and the approved number is IRB00001052-11015. All participants in the study provided written informed consent before participation. The data collected for this study were anonymized, ensuring the protection of participant privacy and adherence to the principles outlined in the Declaration of Helsinki. No harm was inflicted upon the participants during the course of the study.

### Clinical characteristics

Information on sociodemographic characteristics, health behavior variables, anthropometric indicators, medical history and medication usage of participants was collected by trained staffs via standard process^23^. Sociodemographic characteristics included age, gender, education level (primary school or lower, secondary school, higher), marriage status (married, divorced, widowed) and per capita household consumption. Health-related behavior indicators included smoke (never, former, current), drink (never, light: less than once a month and once a month, moderate: less than once a week and once a week, high: more than once a week) and sleep duration (short: less than 6 h, moderate: 6 to 8 h, long: more than 8 h). Anthropometric indexes included weight, height, waist circumference, body mass index (BMI), pulse, systolic blood pressure (SBP) and diastolic blood pressure (DBP). Blood pressure (BP) was measured three times with the individuals in the sitting posture with 45 s apart, and the average of the last two measurements was employed. Medical history included hypertension and diabetes. Hypertension was defined as SBP ≥ 140 mmHg, and/or DBP ≥ 90 mmHg, and/or self‐reported physician's diagnosis, and/or currently using antihypertensive drugs, and/or under other related therapeutic measures. Diabetes was defined by a FBG > 125 mg/dL, and/or a hemoglobin A1c > 6.5%, and/or a self-reported prior diagnosis of diabetes by a doctor, and/or an antidiabetic medication. Medication usage included hypoglycemic drugs and lipid-lowering drugs.

### Laboratory assays

Venous blood samples were collected on an empty stomach at the baseline and were transferred to the Chinese Center for Disease Control and Prevention with a cold-chain management for further analysis^25^. White blood cells (WBC), mean corpuscular volume (MCV), hematocrit, hemoglobin, platelet, blood urea nitrogen (BUN), fasting plasma glucose (FBG), creatine, total cholesterol, triglycerides, high-density lipoprotein cholesterol (HDL-C), low-density lipoprotein cholesterol (LDL-C), hemoglobin A1c (HbA1c), uric acid, C-reactive protein (CRP) were determined by trained research staffs. The TyG index was developed according to the formula ln [fasting triglycerides (mg/dL) × fasting glucose (mg/dL)/2]^26^.

### Definition

The diagnose of central obesity was defined according to the size of the waist circumstance. Normal waist was defined by waist circumstance < 85 cm in men and waist circumstance < 80 cm in women. Central obesity prophase was defined by 85 ≤ waist circumstance < 90 cm in men and 80 ≤ waist circumstance < 85 cm in women. Central obesity was defined by waist circumstance ≥ 90 cm in men and waist circumstance ≥ 85 cm in women.

### Statistical analyses

Continuous variables were listed as mean ± standard deviation (SD) or the median (25th percentile-75th percentile). The number of cases and proportions (%) were presented for categorical variables. One-way analysis of variance (ANOVA) tests or Kruskal Wallis test were used to compare the differences for continuous variables in baseline characteristics. Chi-square tests or Fisher’s exact test were used for categorical variables. Cox proportional hazard model was employed to examine the relationship of TyG and hypertension with adjustments for the above pertinent variables in three models. Model 1 was adjusted for gender, age, smoke, drink, marital status, education level, sleep duration, BMI, diabetes, WBC, PLT, MCV, hemoglobin, hematocrit, CRP, BUN, creatine, uric acid, HDL-C, LDL-C, pulse, use of hypoglycemic drugs, use of lipid-lowering drugs and per capita household consumption; Model 2 was further adjusted for SBP and DBP. In addition, penalized spline method (smooth fitting curve) was further used to explore the relation of TyG with hypertension in groups with different waist circumstance.

In further exploratory subgroup analyses, potential effect modifications of the relationship of TyG with incidence of hypertension in groups with different waist circumstance were assessed according to gender, BMI (< 24 vs. ≥ 24), age (< 60 vs. ≥ 60 years), drink (never vs. light vs. moderate vs. high), smoke (never vs. Former vs. current) and diabetes (no vs. yes).

All data analysed was using the R software version 4.1.0 (www.R-project.org) and EmpowerStates (www.empowerstats.com, X&Y Solutions, Inc., Boston, MA). A 2-tailed *P* < 0.05 was considered statistically significant.

### Ethical approval

Each participant included in this study signed a written informed consent form before taking the survey. Ethics approval for the data collection in CHARLS was obtained from the Biomedical Ethics Review Committee of Peking University (IRB00001052-11015).

## Result

### Baseline characteristics

A total of 5865 eligible participants (2759 males and 3106 females) aged ≥ 45 years old were enrolled in the study. Basic demographic and clinical characteristics of the participants are presented in Table 1. Overall, the mean (SD) age was 56.69 (8.31) years and 47.04% were males. The mean ± SD of baseline triglycerides, FBG, TyG and waist circumstance were 123.64 ± 91.24 mg/dL, 106.59 ± 30.28 mg/dL, 8.60 ± 0.63 and 82.15 ± 11.83 cm, respectively. Individuals in higher quartiles tended to be female, and have a higher rate of diabetes, use of hypoglycemic drugs, use of lipid-lowering drugs, new-onset hypertension; to have higher waist, BMI, WBC, FBG, creatine, total cholesterol, triglycerides, HbA1c, uric acid, SBP, DBP, pulse. No significant differences were found in terms of age, education level, drink, marital status, CRP and sleep duration between the quartiles of TyG index.

**Table 1:** Baseline characteristics of study participants according to tertiles of triglyceride glucose (TyG) index.

TyG quartiles	Total	Q1 (4.96–8.18)	Q2 (8.18–8.52)	Q3 (8.52–8.95)	Q4 (8.95–12.14)	*P* value
N	5865	1466	1466	1466	1467	
Age, years	56.69 ± 8.31	56.70 ± 8.54	56.81 ± 8.54	56.80 ± 8.11	56.45 ± 8.03	0.631
Gender, n (%)						< 0.001
Male	2759 (47.04%)	778 (53.07%)	705 (48.09%)	636 (43.38%)	640 (43.63%)	
Female	3106 (52.96%)	688 (46.93%)	761 (51.91%)	830 (56.62%)	827 (56.37%)	
Education level						0.565
Primary or lower	3840 (65.47%)	966 (65.89%)	973 (66.37%)	961 (65.55%)	940 (64.08%)	
Secondary	1816 (30.96%)	453 (30.90%)	440 (30.01%)	459 (31.31%)	464 (31.63%)	
Higher	209 (3.56%)	47 (3.21%)	53 (3.62%)	46 (3.14%)	63 (4.29%)	
Per capita household consumption, yuan/year	4940 (2896–8651)	4740 (2739–8475)	4885 (2904–8628)	4921 (2944–8440)	5235(3010–9129)	0.034
Smoke, n (%)						0.005
Never	3555 (62.16%)	838 (58.64%)	889 (62.12%)	907 (63.16%)	921 (64.72%)	
Former	414 (7.24%)	106 (7.42%)	97 (6.78%)	95 (6.62%)	116 (8.15%)	
Current	1750 (30.60%)	485 (33.94%)	445 (31.10%)	434 (30.22%)	386 (27.13%)	
Drink, n (%)						0.233
Never	3828 (69.70%)	903 (66.40%)	952 (69.69%)	993 (71.59%)	980 (71.07%)	
Light	579 (10.54%)	158 (11.62%)	142 (10.40%)	140 (10.09%)	139 (10.08%)	
Moderate	254 (4.62%)	65 (4.78%)	63 (4.61%)	61 (4.40%)	65 (4.71%)	
High	831 (15.13%)	234 (17.21%)	209 (15.30%)	193 (13.91%)	195 (14.14%)	
Marital status, n (%)						0.741
Married	5372 (91.61%)	1347 (91.88%)	1337 (91.20%)	1347 (91.95%)	1341 (91.41%)	
Divorced	62 (1.06%)	19 (1.30%)	18 (1.23%)	11 (0.75%)	14 (0.95%)	
Widowed	430 (7.33%)	100 (6.82%)	111 (7.57%)	107 (7.30%)	112 (7.63%)	
Diabetes, n (%)	767 (13.08%)	41 (2.80%)	91 (6.21%)	155 (10.57%)	480 (32.72%)	< 0.001
Waist, cm	82.15 ± 11.83	79.02 ± 10.71	81.37 ± 11.11	82.87 ± 11.33	85.50 ± 13.16	< 0.001
BMI, kg/m^2^	22.57 (20.52–24.84)	21.52 (19.73–23.61)	22.29 (20.21–24.36)	22.65 (20.65–25.01)	24.08 (21.95–26.30)	< 0.001
WBC, 10^9^/L	6.13 ± 1.80	5.92 ± 1.79	5.98 ± 1.78	6.16 ± 1.75	6.45 ± 1.82	< 0.001
MCV, fL	90.41 ± 8.62	90.57 ± 9.13	90.07 ± 8.89	90.53 ± 8.64	90.48 ± 7.75	< 0.001
Hematocrit, %	41.27 ± 6.23	40.97 ± 6.32	40.95 ± 6.23	41.29 ± 5.99	41.87 ± 6.33	< 0.001
Hemoglobin, g/L	14.33 ± 2.19	14.18 ± 2.32	14.21 ± 2.14	14.32 ± 2.08	14.61 ± 2.21	< 0.001
PLT, 10^9^/L	209.97 ± 71.20	209.27 ± 71.11	207.21 ± 70.73	209.37 ± 72.13	214.05 ± 70.71	< 0.001
BUN, mg/dL	15.62 ± 4.36	16.30 ± 4.65	15.72 ± 4.28	15.13 ± 4.17	15.33 ± 4.22	< 0.001
FBG, mg/dL	106.59 ± 30.28	93.99 ± 13.56	100.19 ± 13.40	104.66 ± 17.24	127.49 ± 48.66	< 0.001
Creatine, mg/dL	0.76 ± 0.17	0.75 ± 0.16	0.76 ± 0.17	0.77 ± 0.17	0.78 ± 0.18	0.002
Total cholesterol, mg/dL	190.93 ± 37.26	177.78 ± 33.26	187.41 ± 32.66	194.19 ± 36.35	204.31 ± 41.05	< 0.001
Triglycerides, mg/dL	123.64 ± 91.24	59.09 ± 13.08	86.49 ± 13.69	120.40 ± 21.39	228.51 ± 126.17	< 0.001
HDL-C, mg/dL	52.05 ± 15.24	60.77 ± 15.07	55.08 ± 13.76	50.37 ± 13.43	42.00 ± 11.96	< 0.001
LDL-C, mg/dL	115.36 ± 33.45	107.67 ± 28.96	117.35 ± 29.80	121.87 ± 32.91	114.54 ± 39.53	< 0.001
HbA1c, %	5.21 ± 0.73	5.06 ± 0.41	5.12 ± 0.49	5.17 ± 0.55	5.51 ± 1.15	< 0.001
Uric acid, mg/dL	4.32 ± 1.17	4.16 ± 1.07	4.22 ± 1.15	4.32 ± 1.17	4.57 ± 1.25	< 0.001
CRP, mg/L	0.88 (0.50–1.83)	0.76 (0.44–1.64)	0.79 (0.46–1.65)	0.86 (0.50–1.80)	1.12 (0.62–2.19)	0.919
SBP, mmHg	117.31 ± 11.61	115.49 ± 11.65	116.60 ± 11.67	117.81 ± 11.39	119.43 ± 11.38	< 0.001
DBP, mmHg	69.99 ± 8.85	68.47 ± 8.61	69.63 ± 9.13	70.66 ± 8.86	71.29 ± 8.54	< 0.001
Pulse, times/min	71.80 ± 10.10	70.28 ± 10.03	71.39 ± 9.81	72.60 ± 10.23	73.01 ± 10.12	< 0.001
TyG index	8.60 ± 0.63	7.89 ± 0.27	8.35 ± 0.10	8.72 ± 0.12	9.44 ± 0.47	< 0.001
Use of hypoglycemic drugs, n (%)	120 (2.07%)	11 (0.76%)	17 (1.17%)	20 (1.39%)	72 (4.97%)	< 0.001
Use of lipid-lowering drugs, n (%)	138 (2.40%)	20 (1.38%)	27 (1.87%)	29 (2.04%)	62 (4.32%)	< 0.001
Sleep duration, n (%)						0.442
Short	1583 (28.39%)	383 (27.32%)	402 (28.73%)	427 (30.43%)	371 (27.06%)	
Moderate	2323 (41.67%)	598 (42.65%)	578 (41.32%)	557 (39.70%)	590 (43.03%)	
Long	1669 (29.94%)	421 (30.03%)	419 (29.95%)	419 (29.86%)	410 (29.91%)	
New-onset hypertension, n (%)	1594 (27.18%)	354 (24.15%)	397 (27.08%)	387 (26.40%)	456 (31.08%)	< 0.001

Data are expressed as mean ± standard deviation or median (interquartile range) and numbers (percentage) as appropriate.

*BMI* body mass index, *BUN* blood urea nitrogen, *CRP* C-reactive protein, *DBP* diastolic blood pressure, *FBG* fasting blood glucose, *HbA1c* hemoglobin A1c, *HDL-C* high-density lipoprotein cholesterol, *LDL-C* low-density lipoprotein cholesterol, *MCV* mean corpuscular volume, *PLT* platelet, *SBP* systolic blood pressure, *TyG* triglyceride glucose index, *WBC* white blood cell.

### Association between the TyG and risk of hypertension

**Figure 2:**
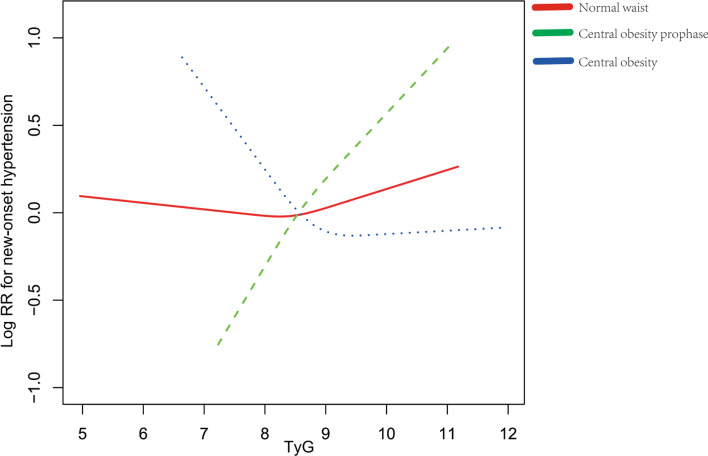
Adjusted cubic spline model of the relationship of baseline TyG with risk of new-onset hypertension. The solid line and dashed line represent the estimated values and their corresponding 95% confidence interval. Adjustment factors included age, gender, smoke, drink, marital status, education level, sleep duration, BMI, diabetes, WBC, PLT, MCV, hemoglobin, hematocrit, CRP, BUN, creatine, uric acid, HDL-C, LDL-C, pulse, use of hypoglycemic drugs, use of lipid-lowering drugs, per capita household consumption, SBP and DBP. Abbreviations: BMI, body mass index; BUN, blood urea nitrogen; CRP, C-reactive protein; DBP, diastolic blood pressure; FBG, fasting blood glucose; HbA1c, hemoglobin A1c; HDL-C, high-density lipoprotein cholesterol; LDL-C, low-density lipoprotein cholesterol; MCV, mean corpuscular volume; PLT, platelet; RR, relative risk; SBP, systolic blood pressure; TyG, triglyceride glucose index; WBC, white blood cell.

The results of the cox proportional hazard models analyzing the hazard ratio (HR) in each TyG group are presented in Table S1. When TyG was analyzed as a continuous variable or a classified variable, the correlation between TyG index and new-onset hypertension was distinctive even after adjustment for pertinent variables. Moreover, a stratified analysis was conducted evaluate potential interactive factors between TyG and the risk of hypertension in various subgroups (Fig. S1). None of the covariates, including gender, age, BMI, drink, smoke and diabetes, notably changed the correlation between TyG and the risk of hypertension (*P*-interaction > 0.05) excluding waist circumstance (*P*-interaction = 0.03). Moreover, the specific relationship was explored in groups with different waist circumstance (Table 2). In individuals with central obesity prophase, compared with the first TyG group (Q1, 4.96–8.18), the HR (95% CI) for individuals in Q3 (8.52–8.95) and Q4 (8.95–12.14) were 1.63 (0.98, 2.71) and 2.03 (1.17, 3.54) after adjustment for pertinent variables, respectively (*P* for trend < 0.01). However, an inverse correlation was found in subjects with central obesity. In the group, the adjusted HR values for participants in the Q3 and Q4 were 0.66 (95% CI 0.47–0.92) and 0.64 (95% CI 0.45–0.91) with TyG assessed in quartiles. In addition, the relationship of TyG with new-onset hypertension in participants with normal waist circumstance remained inconspicuous. Penalized spline method was used to confirm the linearly positive association between the TyG and the risk of hypertension in subjects with central obesity prophase, the negative linear association in subjects with central obesity and inconspicuous association in subjects with normal waist (Fig. 2).

**Table 2:** Hazard ratios of triglyceride glucose (TyG) index for hypertension in participants with different waist circumstance.

Variable	N	HR (95% CI) *P* value		
		Crude model	Model I	Model II
Normal waist				
TyG	2429	1.10 (0.95, 1.28) 0.20	1.11 (0.90, 1.37) 0.34	1.05 (0.84, 1.30) 0.68
TyG quartiles				
Q1 (4.96–8.18)	832	1	1	1
Q2 (8.18–8.52)	674	1.09 (0.88, 1.36) 0.41	1.16 (0.91, 1.49) 0.24	1.13 (0.88, 1.45) 0.33
Q3 (8.52–8.95)	555	1.13 (0.90, 1.41) 0.29	1.02 (0.77, 1.36) 0.89	0.93 (0.69, 1.24) 0.61
Q4 (8.95–12.14)	368	1.05 (0.81, 1.36) 0.70	1.15 (0.81, 1.63) 0.44	1.07 (0.75, 1.52) 0.72
P for trend		0.49	0.61	0.93
Central obesity prophase				
TyG	886	1.11 (0.91, 1.35) 0.29	1.64 (1.18, 2.27) < 0.01	1.57 (1.13, 2.16) < 0.01
TyG quartiles				
Q1 (4.96–8.18)	180	1	1	1
Q2 (8.18–8.52)	230	1.33 (0.90, 1.96) 0.15	1.55 (0.95, 2.52) 0.08	1.37 (0.84, 2.23) 0.21
Q3 (8.52–8.95)	241	1.17 (0.79, 1.73) 0.44	1.80 (1.08, 2.98) 0.02	1.63 (0.98, 2.71) 0.06
Q4 (8.95–12.14)	235	1.48 (1.01, 2.16) 0.04	2.34 (1.35, 4.06) < 0.01	2.03 (1.17, 3.54) 0.01
P for trend		< 0.01	< 0.01	< 0.01
Central obesity				
TyG	1592	1.07 (0.94, 1.21) 0.31	0.92 (0.75, 1.12) 0.41	0.84 (0.69, 1.03) 0.10
TyG quartiles				
Q1 (4.96–8.18)	240	1	1	1
Q2 (8.18–8.52)	331	1.10 (0.82, 1.48) 0.51	0.84 (0.59, 1.18) 0.30	0.76 (0.54, 1.07) 0.12
Q3 (8.52–8.95)	429	1.01 (0.76, 1.34) 0.96	0.72 (0.51, 1.01) 0.05	0.66 (0.47, 0.92) 0.01
Q4 (8.95–12.14)	592	1.12 (0.86, 1.46) 0.40	0.72 (0.51, 1.03) 0.07	0.64 (0.45, 0.91) 0.01
P for trend		0.52	0.07	0.02

Adjust I model adjust for: age, gender, smoke, drink, marital status, education level, sleep duration, BMI, diabetes, WBC, PLT, MCV, hemoglobin, hematocrit, CRP, BUN, creatine, uric acid, HDL-C, LDL-C, pulse, use of hypoglycemic drugs, use of lipid-lowering drugs and per capita household consumption; Adjust II model adjust for: age, gender, smoke, drink, marital status, education level, sleep duration, BMI, diabetes, WBC, PLT, MCV, hemoglobin, hematocrit, CRP, BUN, creatine, uric acid, HDL-C, LDL-C, pulse, use of hypoglycemic drugs, use of lipid-lowering drugs, per capita household consumption, SBP and DBP.

*BMI* body mass index, *BUN* blood urea nitrogen, *CRP* C-reactive protein, *CI* confidence interval, *DBP* diastolic blood pressure, *FBG* fasting blood glucose, *HbA1c* hemoglobin A1c, *HDL-C* high-density lipoprotein cholesterol, *HR* hazard ratio, *LDL-C* low-density lipoprotein cholesterol, *MCV* mean corpuscular volume, *PLT* platelet, *SBP* systolic blood pressure, *TyG* triglyceride glucose index, *WBC* white blood cell.

### Subgroup analysis

**Figure 3:**
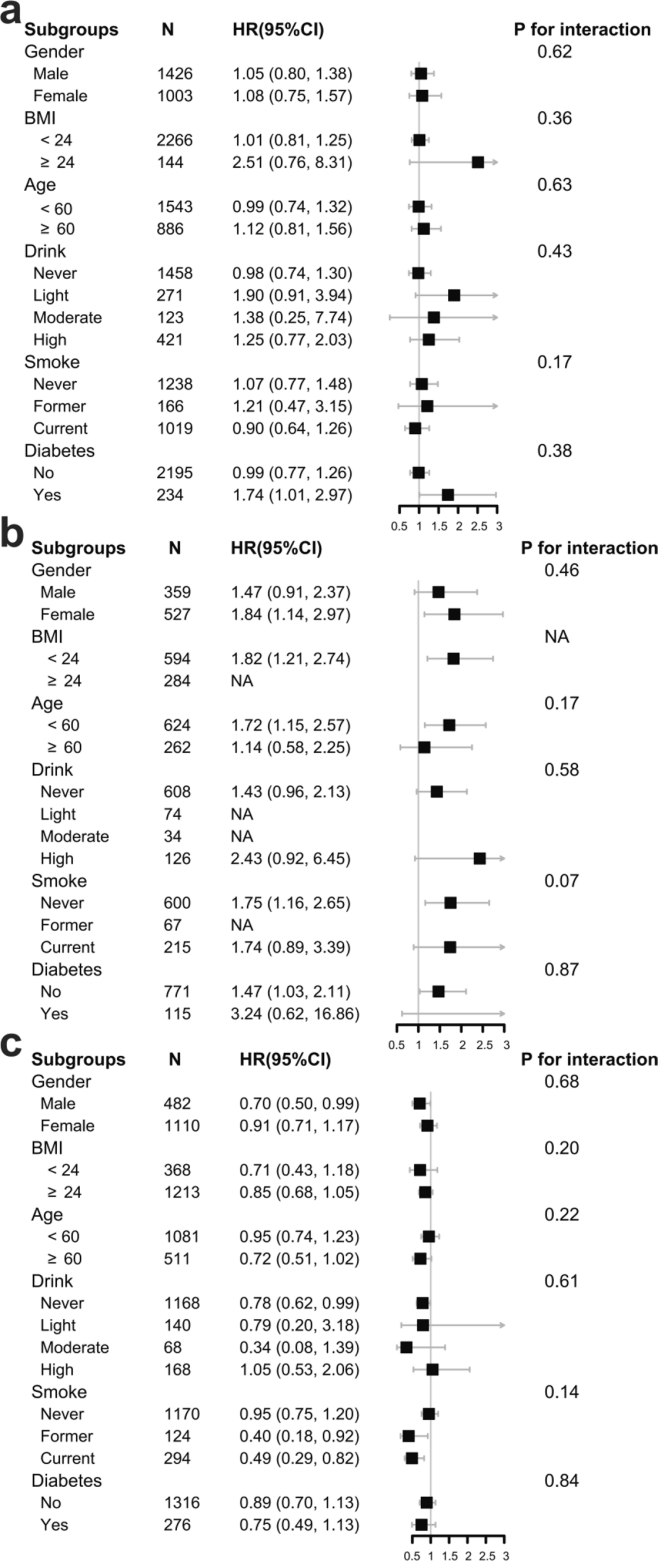
Subgroup analyses of the association between TyG index and new-onset hypertension in individuals with normal waist (**a**), central obesity prophase (**b**) and central obesity (**c**). The multivariate model was adjusted for age, gender, smoke, drink, marital status, education level, sleep duration, BMI, diabetes, WBC, PLT, MCV, hemoglobin, hematocrit, CRP, BUN, creatine, uric acid, HDL-C, LDL-C, pulse, use of hypoglycemic drugs, use of lipid-lowering drugs, per capita household consumption, SBP and DBP, with the exception of the variable that was stratified. Subgroup for diabetes was adjusted for age, gender, smoke, drink, marital status, education level, sleep duration, BMI, WBC, PLT, MCV, hemoglobin, hematocrit, CRP, BUN, creatine, uric acid, HDL-C, LDL-C, pulse, use of lipid-lowering drugs, per capita household consumption, SBP and DBP. Abbreviations: BMI, body mass index; BUN, blood urea nitrogen; CRP, C-reactive protein; DBP, diastolic blood pressure; FBG, fasting blood glucose; HbA1c, hemoglobin A1c; HDL-C, high-density lipoprotein cholesterol; LDL-C, low-density lipoprotein cholesterol; MCV, mean corpuscular volume; PLT, platelet; SBP, systolic blood pressure; TyG, triglyceride glucose index; WBC, white blood cell.

Further stratified analyses were conducted to determine the relationship of TyG with risk of hypertension for individuals with different waist circumstances in various subgroups (Fig. 3). The associations between TyG index and new-onset hypertension were consistent for all groups with different waist in the following subgroups: gender (male vs. female; *P* for interaction = 0.62, 0.46, 0.68), BMI (< 24 vs. ≥ 24 kg/m^2^; *P* for interaction = 0.36, NA, 0.20), age (< 60 vs. ≥ 60; *P* for interaction = 0.63, 0.17, 0.22), drink (never, light, moderate, high; *P* for interaction = 0.43, 0.58, 0.61), smoke (never, former, current; *P* for interaction = 0.17, 0.07, 0.14) and diabetes (no vs. yes; *P* for interaction = 0.38, 0.87, 0.84).

## Discussion

This prospective cohort study demonstrated that there was a positive relationship of TyG with the risk of hypertension in middle-aged and elderly Chinese population with central obesity prophase, a negative correlation of TyG with hypertension in individuals with central obesity and inconspicuous association in subjects with normal waist circumstance.

Most previous studies supported the function of TyG as a predictive marker for hypertension. Zhang et al.^17^ indicated that increased TyG was significantly related to prehypertension and hypertension in normoglycemic population across a large cross-sectional study. Furthermore, a meta-analysis including 200,044 participants of eight observational studies^27^ reported that compared with the lowest TyG quartile, the highest TyG quartile was associated with a 53% higher risk of hypertension [risk ratio (RR): 1.53, 95% CI 1.26–1.85, *P* < 0.001]. A prospective 9-year cohort study of 4686 Chinese healthy subjects showed that high baseline TyG was an independent risk for developing hypertension^15^. There were several literatures investigating the interaction of obesity index on the relationship of TyG with hypertension, but seldom research found the joint effect of waist circumstance groupings. For instance, Liu et al.^28^ showed that TyG was significantly associated with hypertension in group with normal BMI, while the association was not statistically significant in participants with elevated BMI. Moreover, another cross-sectional study including middle-aged and elderly adults revealed that there was significant interaction between TyG index and WHtR (waist-to-height ratio) on risk of hypertension^16^. A study^29^ including 105,070 lean adults without hypertension demonstrated that TyG-BMI [AUC (area under the curve): 0.619 (95% CI 0.614–0.625)] and TyG-waist circumstance [AUC: 0.618 (95% CI 0.612–0.623)] were notably related to prehypertension, and the AUC was much higher than that of TyG [0.605 (95% CI 0.600–0.611)]. Additionally, in a population-based cross-sectional study conducted by Wang et al.^30^ among 2076 Chinese subjects in Henan province, the significant additive interactions of TyG and WHtR, percent body fat hypertension risk were observed.

There are some new insights provided into the filed in the work. First, there was inconspicuous association of TyG with risk of hypertension in individuals with normal waist, which is inconsistent with previous studies. This could be explained by the close relationship between glucose, triglyceride and waist. In fact, subjects with persistent increments of blood glucose and/or triglyceride tended to have a higher waist circumstance. Therefore, it is reasonable to speculate that the elevated TyG in individuals with normal waist should be transient and has no prognostic value. Second, dose–response association of TyG with hypertension was only found in individuals with central obesity prophase. As waist circumference increases, visceral fat is accumulated, contributing to metabolic syndrome, insulin resistance, elevated blood pressure, and activation of the sympathetic nerve system^31^. On that basis, elevated TyG may indicate the more severe degree of insulin resistance meaning a higher risk of hypertension. Third, there was a negative relationship of TyG index with hypertension in subjects with central obesity. The inconsistent finding that relative risk of hypertension in middle-aged and elderly Chinese individuals gradually decreased with increasing baseline TyG index in subjects with central obesity also raises questions about the widespread application of TyG values. Possible explanation for this difference including (1) There is a readily recognizable phenotype associated with abdominal obesity that is believed to reflect an increased cardiovascular risk, and the population may receive or seek changes in lifestyle earlier prior to the onset of hypertension, thereby changing the natural course of the disease compared with thin persons^32^; (2) Generally speaking, as a population with a high prevalence of hypertension, prolonged exposure to central obesity and elevated TyG should produce a cumulative effect of onset of hypertension for middle-aged and elderly adults. However, hypertensive population has been screened out for the research design. Other factors being equal, the higher baseline TyG index may instead indicate that there are less hypertension-susceptibility genes carried in the central obesity population without onset of hypertension^33–35^. This doesn’t go against the evidence that central obesity and elevated TyG are risk factors for hypertension. These findings challenged the view that TyG may be of universal use in evaluating the risk of hypertension in clinical practice. The research acknowledges that the TyG becomes an indicator of hypertension for middle-age and elderly Chinese adults only when combined with the unique grouping by measurement of waist circumference.

Although the specific mechanisms underlying the correlation of the TyG with hypertension, especially in populations with normal waist or central obesity, is not yet clear, TyG may predict hypertension by reflecting the level of IR. IR may exert an influence on hypertension by activating the sympathetic nervous system. The insulin abnormalities induced by IR are particularly pronounced in obese individuals and those with type 2 diabetes, leading to an excessive activation of the sympathetic nervous system. The heightened activity of the sympathetic nervous system can result in vasoconstriction, increased cardiac output, and fluid retention, ultimately contributing to elevated blood pressure^36^. Furthermore, IR may also enhance the activity of the RAAS, a pivotal system in maintaining fluid balance and blood pressure. IR may lead to an overactivation of RAAS, triggering physiological effects that contribute to an increase in blood pressure^37^. Additionally, the impact of IR on the kidneys may further exacerbate hypertension by promoting the reabsorption of sodium. The increased reabsorption of sodium leads to fluid retention, increasing blood volume and consequently elevating blood pressure. This process may be attributed to the direct effects of insulin on renal tubules, influencing sodium channels and transporters. In addition, IR may augment the role of modifiable risk factors of hypertension, such as hyperinsulinaemia, hyperglycaemia, dyslipidemia, a proinflammatory state and arterial stiffness^26,38–40^. Obesity itself is already associated with the activation of the sympathetic nervous system. In the context of obesity, the interaction between insulin resistance and the sympathetic nervous system may contribute to the emergence of interaction. In the study, there was a prominent interaction effect of waist circumstance rather than BMI in the subgroup analyses evaluating the relationship of TyG with hypertension. Compared with BMI, waist circumstance seems to offer more advantages over central arterial stiffness, abnormalities of the hypothalamic–pituitary–adrenal axis and dysfunctional adipose tissue^41–43^, all of which predispose to risk of hypertension.

## Study strengths and limitations

This cohort study has many strengths and some limitations. The strengths of the present study were the inclusion of a large scale of nonhypertensive middle-aged and elderly Chinese participants, adjustment of potential confounding elements such as sociodemographic characteristics, health behavior variables, anthropometric indicators and laboratory assays to minimize residual confounders, handled target independent variables as both continuous variables and categorical variables to reduce the contingency in the data analysis, and the subgroup analyses. In addition, this study also determined the difference in the TyG in predicting hypertension according to waist circumstance. The study has several limitations that warrant acknowledgement.

First, the study individuals were from middle-age and elderly Chinese adults and the grouping by waist circumstance was only according to criteria of Chinese population; thus, these findings may cannot be extrapolated to other populations. Second, the study only investigated the relationship of baseline TyG index with hypertension; dynamic changes over time of TyG index during follow-up remained to be explored. Third, it has been hypothesized that the less hypertension-susceptibility genes carried in the central obesity population without onset of hypertension might account for the negative trend observed between TyG and hypertension; however, the detailed gene-specific mechanism is limited. Fourth, the roles of the sympathetic nervous system and the RAAS in this context remain speculative and warrant further exploration. Finally, potential residual confounding might still exist despite adjustments for pertinent factors.

## Conclusion

In conclusion, the current study examined whether waist circumstance modified the relationship between TyG index and hypertension among middle-aged and elderly Chinese adults. These findings suggested the positive association of TyG with risk of hypertension among population with central obesity prophase, negative association of TyG with hypertension among population with central obesity and inconspicuous correlation of TyG with hypertension among population with normal waist. Therefore, TyG, as a simple and effective risk assessment indicator, should be taken into account in hypertension management in clinical practice when combined with measurement of waist circumference in middle-aged and elderly Chinese adults.

### Supplementary Information

Supplementary Information.

## Data Availability

This data was extracted from the CHARLS website (https://charls.pku.edu.cn/) and the Harmonized CHARLS (https://charls.charlsdata.com/pages/Data/harmonized_charls/en.html).

## Abbreviations

IR: Insulin resistance
TyG: Triglyceride glucose index
RAAS: Renin–angiotensin–aldosterone system
CHARLS: China Health and Retirement Longitudinal Study
BMI: Body mass index
BP: Blood pressure
SBP: Systolic blood pressure
DBP: Diastolic blood pressure
WBC: White blood cell
MCV: Mean corpuscular volume
BUN: Blood urea nitrogen
FBG: Fasting plasma glucose
HDL-C: High-density lipoprotein cholesterol
LDL-C: Low-density lipoprotein cholesterol
HbA1c: Hemoglobin A1c
CRP: C-reactive protein
SD: Standard deviation
ANOVA: Analysis of variance
HR: Hazard ratio
RR: Risk ratio
WHtR: Waist-to-height ratio
AUC: Area under the curve
