# Supplementary material for: Association of TyG index and central obesity with hypertension in middle-aged and elderly Chinese adults: a prospective cohort study
Source: Sci Rep. 2024 Jan 26;14:2235. doi: 10.1038/s41598-024-52342-7 (PMC10817920; doi:10.1038/s41598-024-52342-7)
Supplement: Supplementary file 1 — Supplementary Information. [file 41598_2024_52342_MOESM1_ESM.docx]

| Table S1. Cox-proportional hazard models for the association between triglyceride glucose (TyG) index and incident hypertension. | | | | |
| --- | --- | --- | --- | --- |
| **Exposure** | **N** | **HR (95% CI) *P*-value** | | |
|  |  | **Crude model** | **Model Ⅰ** | **Model Ⅱ** |
| TyG | 5865 | 1.09 (1.00, 1.19) 0.06 | 1.11 (0.98, 1.27) 0.10 | 1.03 (0.91, 1.18) 0.62 |
| TyG quartiles |  |  |  |  |
| Q1(4.96-8.18) | 1466 | 1 | 1 | 1 |
| Q2(8.18-8.52) | 1466 | 1.14 (0.97, 1.33) 0.11 | 1.12 (0.93, 1.34) 0.24 | 1.05 (0.88, 1.26) 0.58 |
| Q3(8.52-8.95) | 1466 | 1.08 (0.92, 1.27) 0.34 | 1.02 (0.84, 1.24) 0.85 | 0.93 (0.76, 1.13) 0.44 |
| Q4(8.95-12.14) | 1467 | 1.17 (0.99, 1.37) 0.06 | 1.12 (0.90, 1.39) 0.32 | 0.99 (0.80, 1.24) 0.96 |
| P for trend |  | 0.12 | 0.53 | 0.64 |

Adjust Ⅰ model adjust for: age, gender, smoke, drink, marital status, education level, sleep duration, BMI, waist, diabetes, WBC, PLT, MCV, hemoglobin, hematocrit, CRP, BUN, creatine, uric acid, HDL-C, LDL-C, pulse, use of hypoglycemic drugs, use of lipid-lowering drugs and per capita household consumption; Adjust Ⅱ model adjust for: age, gender, smoke, drink, marital status, education level, sleep duration, BMI, waist, diabetes, WBC, PLT, MCV, hemoglobin, hematocrit, CRP, BUN, creatine, uric acid, HDL-C, LDL-C, pulse, use of hypoglycemic drugs, use of lipid-lowering drugs, per capita household consumption, SBP and DBP.

Abbreviations: BMI, body mass index; BUN, blood urea nitrogen; CRP, C-reactive protein; DBP, diastolic blood pressure; FBG, fasting blood glucose; HbA1c, hemoglobin A1c; HDL-C, high-density lipoprotein cholesterol; LDL-C, low-density lipoprotein cholesterol; MCV, mean corpuscular volume; PLT, platelet; SBP, systolic blood pressure; TyG, triglyceride glucose index; WBC, white blood cell.


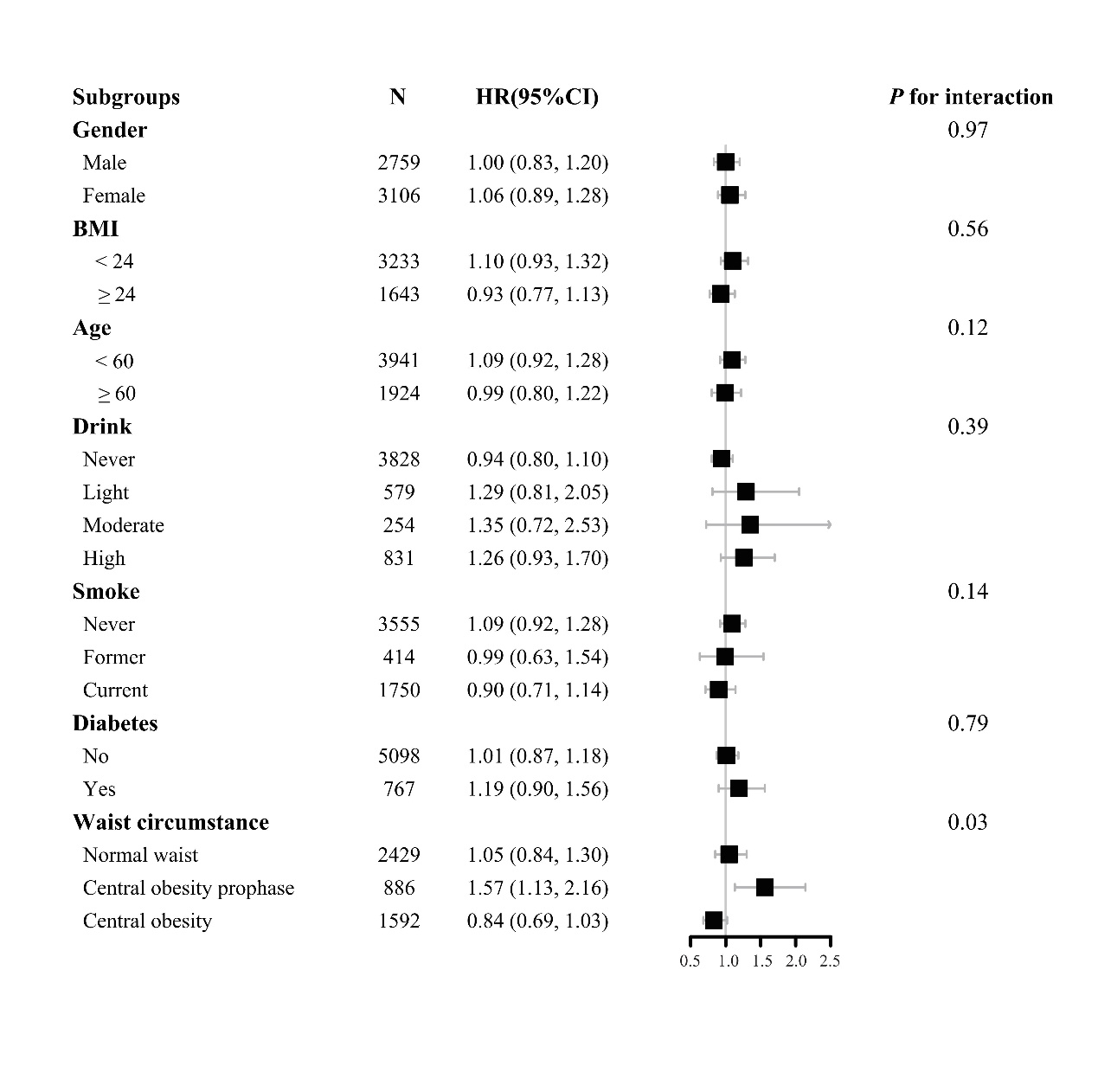


**Figure S1. Subgroup analyses of the association between TyG index and new-onset hypertension.**

The multivariate model was adjusted for age, gender, smoke, drink, diabetes, BMI, BUN, creatine, HDL-C, LDL-C, uric acid, SBP, DBP, lipid-lowering drugs and medications for diabetes.

The multivariate model was adjusted for age, gender, smoke, drink, marital status, education level, sleep duration, BMI, diabetes, WBC, PLT, MCV, hemoglobin, hematocrit, CRP, BUN, creatine, uric acid, HDL-C, LDL-C, pulse, use of hypoglycemic drugs, use of lipid-lowering drugs, per capita household consumption, waist, SBP and DBP, with the exception of the variable that was stratified.

Abbreviations: BMI, body mass index; BUN, blood urea nitrogen; CRP, C-reactive protein; DBP, diastolic blood pressure; FBG, fasting blood glucose; HbA1c, hemoglobin A1c; HDL-C, high-density lipoprotein cholesterol; LDL-C, low-density lipoprotein cholesterol; MCV, mean corpuscular volume; PLT, platelet; SBP, systolic blood pressure; TyG, triglyceride glucose index; WBC, white blood cell.
